# Optimized 2D array of thin silicon pillars for efficient antireflective coatings in the visible spectrum

**DOI:** 10.1038/srep24947

**Published:** 2016-04-25

**Authors:** Julien Proust, Anne-Laure Fehrembach, Frédéric Bedu, Igor Ozerov, Nicolas Bonod

**Affiliations:** 1 Aix-Marseille Université, CNRS, Centrale Marseille, Institut Fresnel, UMR7249, 13013 Marseille, France; 2 Aix-Marseille Université, CNRS, CINAM, UMR 7325, 13288 Marseille, France

## Abstract

Light reflection occuring at the surface of silicon wafers is drastically diminished by etching square pillars of height 110 nm and width 140 nm separated by a 100 nm gap distance in a square lattice. The design of the nanostructure is optimized to widen the spectral tolerance of the antireflective coatings over the visible spectrum for both fundamental polarizations. Angle and polarized resolved optical measurements report a light reflection remaining under 5% when averaged in the visible spectrum for both polarizations in a wide angular range. Light reflection remains almost insensitive to the light polarization even in oblique incidence.

Light reflection occurring at an interface between different materials results from an impedance mismatch due to a contrast of refractive index. Light reflection depends on the angle of incidence, light polarization and optical contrast between the materials defined as the difference between the refractive indexes. Among the wide variety of materials, silicon and glass have attracted most of the attention for application in optics (glass) and opto-electronics (silicon). In the case of silicon, the optical contrast with air is around 2.5 in the visible and near infrared spectrum, which leads to an important reflectivity. This phenomenon lessens the efficiency of a wide variety of opto-electronic components such as CCD or CMOS sensors or also photovoltaic cells. Different strategies have been proposed to design anti-reflective coatings^1^. A thin dielectric coating was first proposed to add a second interface leading to a destructive interference between the two reflected beams for a given set of parameters such as incidence and frequency^2^,^3^. Thin dielectric multilayers lead to a 1D structuring in the direction normal to the interface. An alternative strategy consists of structuring the interface with 2D planar nanostructures^4^,^5^,^6^,^7^,^8^. This method is very interesting since it does not require different materials and the versatile shape of the nanostructuring offers additional optimizing parameters for better performances. A wide variety of 2D nanostructures have been proposed to design antireflective coatings such as nanowires, nanodomes or nanopyramids^9^,^10^,^11^,^12^,^13^,^14^,^15^. Biomimetic arrays inspired by the geometric arrangement of the insect compound eyes^5^,^16^,^17^ and biperiodic arrays of silicon particles were one of the first structures proposed to design anti-reflective coatings^18^,^19^,^20^,^21^. Adding biperiodic arrays on front and back interfaces of thin opaque metallic film yields high light transmission at a given frequency^22^. A highly efficient anti-reflective coating of silicon particles was proposed in 1997 by Lalanne and Morris^23^. By using optical beam lithography, they nanostructured a 100 nm thick silicon layer and showed a strong reduction of light reflection over a broad spectrum and with very weak sensitivity to light polarization. Lastly, the nanoimprint technique was used to etch silicon particles with an aspect ratio close to unity and a period of 450 nm^24^. Emphasize was placed on the resonant properties of individual silicon Mie resonators and on their unique scattering properties due to the excitation of electric and magnetic multipolar resonances^24^,^25^,^26^,^27^,^28^,^29^. Resonant scatterers are good candidate to design thin antireflective coatings^30^. Metallic particles hosting localized surface plasmons can be added to high refractive index to facilitate the light transmission between different optical materials^31^,^32^,^33^. More recently, the growing field of all-silicon metasurfaces attracted a keen interest to control light reflection and transmission through interfaces^34^,^35^,^36^,^37^. Remarkably, it was lastly demonstrated that well designed all-dielectric metasurfaces composed of silicon nanodisks can yield a Brewster effect, *i.e.* a zero of light reflection, at any angle of incidence, frequency and polarization^38^.

Here we report the design, fabrication and characterization of an antireflective thin etched silicon layer that features a wide tolerance over the whole visible spectrum. We consider silicon nanopillars with square cross-sections of width 140 nm and thickness of 110 nm. The objective is to conceive anti-reflective structures featuring a high spectral tolerance together with a weak dependence on the incident polarization. The analysis of the light transmission is performed in the framework of the diffraction grating theory. We analyse the diffraction orders in the whole spectra and reveal that the spectral tolerance results from high diffracted efficiencies in a series of specific diffracted orders. In particular, we show that the impedance matching between air and silicon results from an efficient coupling of the (±1, 0) diffractive orders in silicon by the incident light. We report a light reflection remaining under 5% for both fundamental polarizations (*s* and *p*) for angles of incidence ranging from 0 to 20° in the spectral range [500;900] nm. When considering reflection spectra averaged over the whole visible spectrum, it is showed that the averaged light reflection remains under 5% from 0 to 40° for both polarizations.

## Results

### Design

**Figure 1:**
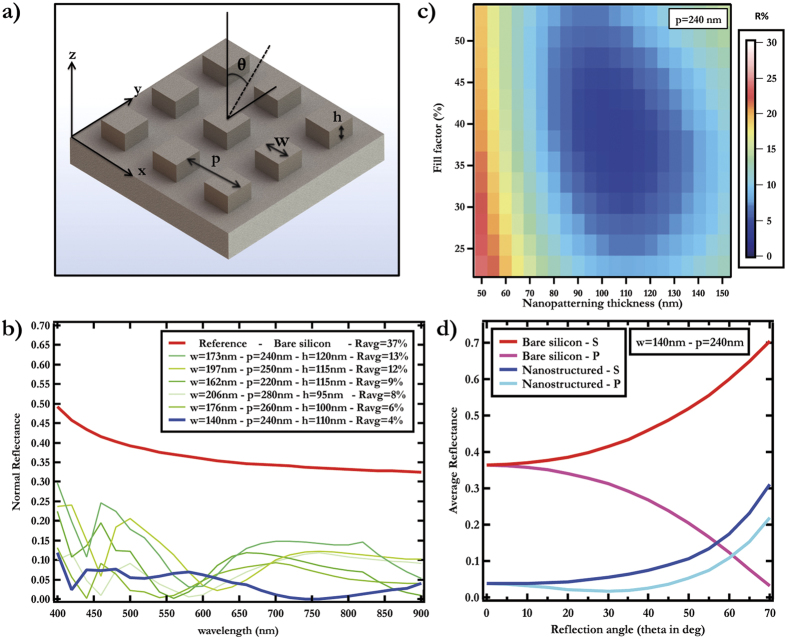
Parametric optimization. (**a**) Sketch of the biperiodic nanostructured anti-reflective coating. A 2D periodic array with a square cell made of Si bumps with a square cross-section of width *w* and height *h* is etched on a silicon substrate. The superstrate is made of air (*n* = 1). The refractive index of silicon is taken from^43^. The period *p* of the grating is the same along the two axes *x* and *y*. (**b**) Reflection of light calculated with respect to the wavelength for a few set of parameters detailed in the inset. (**c**) Reflectivity averaged over the spectral range [400;900] nm calculated with respect to the filling factor (*w*/*p*)^2^ with *p* = 240 nm and height of the Si bumps *h*, when the structure is illuminated from air in normal incidence. (**d**) Reflection averaged over the spectral range [400;900] nm calculated with respect to the angle of incidence for the *s* and *p* polarizations, for the parameters *p* = 240 nm, *h* = 110 nm, and *w* = 140 nm.

The structure under study consists of a silicon substrate on which is engraved a periodic pattern with a square cell. The period *p* is the same in the two in plane directions *x* and *y* (see Fig. 1). The bumps have a square shape with width *w* and depth *h*. The structure is illuminated with a plane wave, the incident wavevector makes an angle *θ* with respect to the normal *z* to the structure, and the plane of incidence is along one direction of periodicity of the grating (*y* axis, see Fig. 1a). The incident wavelength ranges between 400 and 900 nm. The *s* and *p* polarizations correspond respectively to an electric or a magnetic field perpendicular to the plane of incidence.

The optimization of the parameters (period *p*, height *h* and width *w* of the bumps) is performed through a parametric study under normal incidence (*θ* = 0°). The value of the pitch is chosen smaller than the wavelength in order to prevent the propagation of non-null diffraction orders in the superstrate made of air. The period is varied between 200 nm and 400 nm and the depth ranges between 50 nm and 150 nm, which is much smaller than the wavelength. The width is varied between 40 nm and 160 nm. The parametric study is carried out thanks to a home-made numerical code based on the Fourier Modal Method^39^ (see section Methods).

Some of the most interesting simulated reflectivity spectra are represented on Fig. 1b, together with that of the silicon substrate without patterning (red line). We observe a strong diminution of the reflectivity with respect to the non-patterned substrate over the whole visible spectrum for different sets of parameters. For the structure with period *p* = 240 nm, we plot in Fig. 1c the reflectivity averaged over the [400–900] nm range with respect to the thickness *h* and filling factor (ratio of the area covered by the material over the basic cell area (*w*/*p*)^2^. We observe that the minimum reflectivity in the visible spectrum (4% in average) is obtained for parameters close to *w* = 140 nm, and *h* = 110 nm.

Silicon is thereby black due to the near perfect impedance matching between the superstrate made of air and silicon which features optical losses in the visible spectrum. The parametric optimization was performed in normal incidence but finally, we plot in Fig. 1d the reflectivity averaged over the 400–900 nm range for the optimized structure (*p* = 240 nm, *w* = 140 nm, *h* = 110 nm) with respect to the polar angle of incidence *θ*, for the *s* and *p* polarizations (the plane of incidence contains one direction of periodicity of the grating). We observe that the designed anti-reflection coating shows a high tolerance with respect to *θ*, and very importantly, a very weak dependence on the polarization. The reflectivity for the bare silicon is also represented in Fig. 1d for both polarizations.

### Fabrication

**Figure 2:**
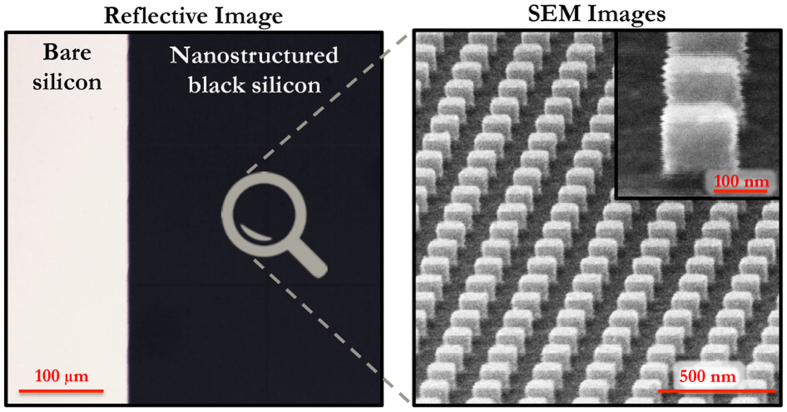
Black silicon. (**a**) Optical image of the bare and nanostructured silicon (with the parameters of Fig. 1d). (**b**) Scanning electron microscopy image (the tilt angle was 65°) of the anti-reflective coating consisting of a 2D periodic array of square silicon particles, 110 nm in height with a square cell.

The anti-reflective coating was fabricated *via* electron beam lithography on 1 cm × 1 cm large (100) oriented monocrystaline silicon wafers (250–300 *μ*m thick). The targeted shape is a square array of silicon bumps, 110 nm in depth, with a period *d* = 240 nm and a width *w* = 140 nm leading to gaps of 100 nm. A 65 nm thick electron beam positive photoresist (PMMA) is first spin-coated before being insolated with an electron beam (Pioneer system, Raith) at a dose around 150 *μ*C.cm^−2^ using 20 kV acceleration voltage. After development, a 20 nm nickel layer is evaporated to form the mask. Unprotected areas are etched by a run composed by a step of a gas mixture (SF_6_ – O_2_) followed by a step of a pure O_2_ plasma exposure. Several run are necessary to etch the targeted silicon thickness. The nickel mask is removed by an aqueous acid solution of FeCl_3_ and the sample is rinsed by deionized water and dried under nitrogen flow (see section methods). Finally, the nanostructured silicon appears black (see Fig. 2a) in the nanostructured area. The scanning electron microscopy reveals the shape and the lattice of the silicon bumps (see Fig. 2b).

### Characterization

**Figure 3:**
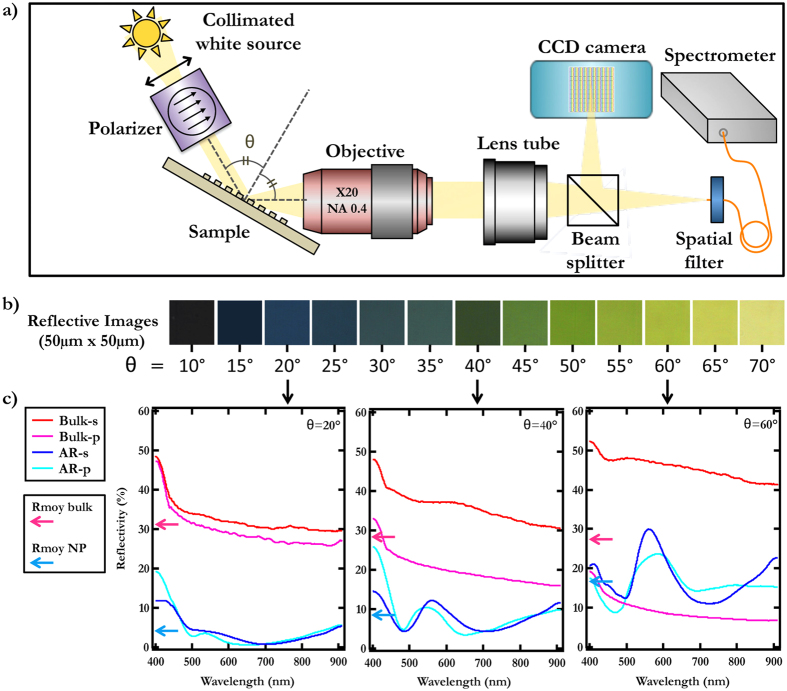
Optical characterization. (**a**) Sketch of the experimental set-up used for monitoring the reflectivity of the sample with respect to the (i) polarization, (ii) angle of incidence and (iii) frequency (see section Methods). (**b**) Optical image of the sample for different angles of incidence. (**c**) Reflectivity measured as function of the wavelength for *s* and *p* polarizations, for the bare and nanostructured silicon, at three angles of incidence (from left to right: 20°, 40°, 60°).

The reflection spectra of the bare and nanostructured silicon samples are then monitored in a home-made confocal microscope (see section Methods). The samples are illuminated with a stabilized halogen lamp beam polarized using a Glan-Thompson linear polarizer (see Fig. 3a). The illuminating beam is placed on a goniometer that can rotate around the sample. The sample can also rotate around its axis that allows us to control the angle of incidence *θ* of the incident beam on the sample and to collect the reflected beam (see Fig. 3a and section Methods). The light reflected by the sample is collected by a ×20 Mitutoyo microscope objective, numerical aperture of 0.4, with a long working distance (20 mm) working with a 200 mm focal lens tube. The reflected beam is spatially filtered with a 105 *μ*m core fiber located at a confocal position to the sample and plugged in a photospectrometer (see Fig. 3a). With this set-up, the reflection spectra of the sample can be measured with respect to the incident wavelength and the angle of incidence for both *s* and *p* polarizations.

Images acquired with the confocal microscope when illuminating the samples with the white source are displayed in Fig. 3b for different angles of incidence. It can be seen that the sample appears black for angles of incidence below 15° and remains very dark up to 40°. The reflection spectra measured at three different angles (*θ* = 20°, *θ* = 40°, *θ* = 60°) are shown in Fig. 3c for the *s* and *p* polarizations and for bare and nanostructured silicon. It can be first observed that the reflection spectra remain almost insensitive to the polarization even for high angles of incidence. As expected, the reflection of the bulk silicon for the p-polarization decreases with incidence since it has a zero at the Brewster incidence near 74°. The reflection averaged over the whole spectrum and the two polarizations is indicated by arrows for the bulk and nanostructured silicon (*R*_*moy*_ bulk and *R*_*moy*_ NP). It can be observed that *R*_*moy*_ NP remains always smaller than *R*_*moy*_ bulk, even for high angles of incidence (60°). The high efficiency of the antireflective coating in the region of interest, usually in the range *θ* = [0;40°] for cameras and photovoltaic cells, is remarkable since for *θ* = 20°, light reflection remains under 5% for both polarizations in the spectral range [500;900] nm (while it is around 30% for the bare silicon).

## Discussions

**Figure 4:**
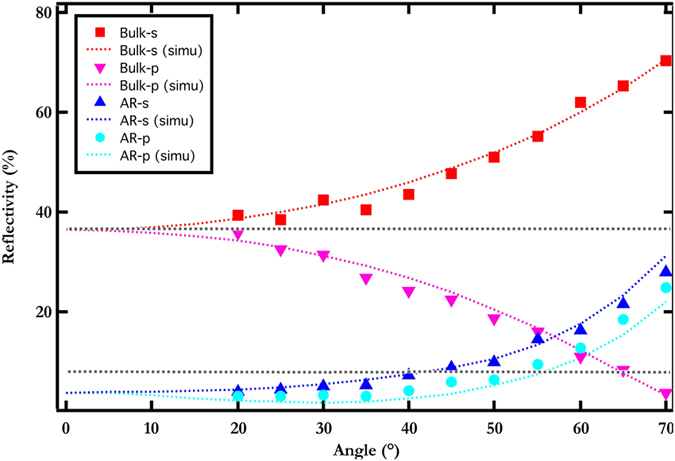
Angular dependence of the spectral averaged reflectivity. Measured reflectivity averaged over the spectral range [400;900] nm, as a function of the angle of incidence for both *s* (blue triangles) and *p* (turquoise circles) polarizations. Comparison with bare silicon, for *s* (red squares) and *p* (pink triangles). Dotted colored lines: simulated results. Dotted grey lines: average reflectivity, top for bare silicon, bottom for structured silicon.

The dependence of the reflection spectra on the angle of incidence is investigated by plotting the reflectivity averaged over the spectral range [400;900] nm with respect to the angle of incidence (see Fig. 4). The measured angular dependence averaged over the visible spectrum is compared with numerical results obtained by the Fourier Modal Method, for both bare and nanostructured silicon and *s*, *p* polarizations. A remarkable concordance between experimental and simulated spectra is observed for the 4 configurations (bare and nanostructured silicon, *s* and *p* polarizations). The strong dependence on the polarization with the bare silicon is drastically weakened with the thin antireflective coating. In the case of pristine silicon, light reflection decreases with the angle of incidence *θ* in *p* polarization due to the Brewster incidence near 74°, while it increases with *θ* in *s* polarization. The very small pitch and very thin bumps added to the bare silicon cancels this trend since it can be observed that light reflection tends to smoothly increase with *θ* for both polarizations. The key result of this study is the fact that the light reflection, averaged over the visible spectrum, remains under 7.5% in the range [0;55°] in *p* polarization and in the range [0;40°] in *s* polarization. Let us emphasize that this wide spectral tolerance together with the very weak sensitivity on the incident polarization are obtained simply by nanostructuring silicon without additional thin film coating. The thickness of the silicon structuring could be further decreased by diminishing the pitch of the structuring and increasing the filling factor of the silicon pillars.

**Figure 5:**
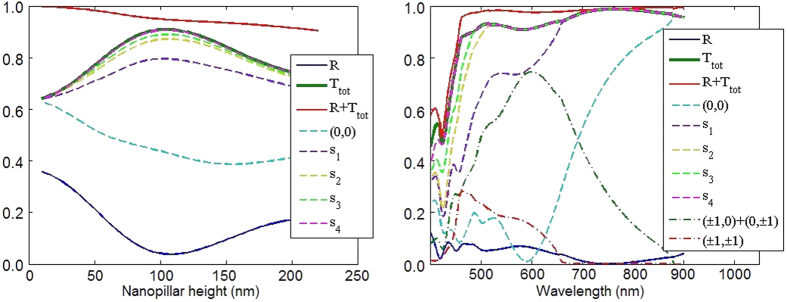
Simulated transmission efficiency. (**a**) Transmission efficiencies averaged over the spectrum with respect to pillar height *h* in nm. Full lines: Reflection (blue), transmission (green), reflection + transmission (red) efficiencies. Dotted lines: transmission efficiency in the (0, 0) order, s_1_: sum of the (0, 0), (0, ±1) and (±1, 0) orders, s_2_: s_1_ + (±1, ±1) orders, s_3_: s_2_ + (0, ±2) + (±2, 0) orders, s_4_: s_3_ + (±1, ±2) + (±2, ±1) orders. (**b**) Transmission efficiencies with respect to the wavelength *λ* in nm for *h* = 110 nm.

To get more insight into the origin of the wide spectral tolerance of the anti-reflective coating, we calculated, for normal incidence, the energy associated to each propagative order in the substrate. We first show in Fig. 5a the transmission efficiency averaged over the spectrum with respect to the pillar height, with the contribution of each propagative order in the substrate (made of Si). The order denoted (*m*, *n*) corresponds to the reciprocal vector 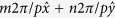. We plot the (0, 0) order that is preponderant in average over the whole spectrum, and then add the different propagative orders in order to highlight their distinct contribution to the transmitted energy. The curve labeled s_1_ is the sum of the (0, 0), (0, ±1) and (±1, 0) orders, s_2_ is calculated taking into account in the sum the four supplementary (±1, ±1) orders, for s_3_ the (0, ±2) and (±2, 0) orders, and for s_4_ the (±1, ±2) and (±2, ±1) orders. We can see that the high spectral averaged transmission observed for *h* = 110 nm results mainly from the contribution of the (0, 0), (0, ±1), (±1, 0) and (±1, ±1) propagative orders. We can also note that the near perfect transmission results from the contribution of all propagative orders between (0, 0) to (±1, ±2) and (±2, ±1). We also plotted in Fig. 5b the different sums of propagative orders s_1_, s_2_, s_3_ and s_4_ with respect to the wavelength for *h* = 110 nm, together with the (0, 0) order only and the sums of the (0, ±1) and (±1, 0) orders only and of the (±1, ±1) only. This allows us to see that the (0, 0) order is preponderant in the spectral range [700 nm;900 nm], that the (0, ±1) (±1, 0) orders are preponderant in the range [500 nm;700 nm], while the higher orders (±1, ±1) permit to achieve a high transmission for wavelengths between 450 nm and 600 nm. These results evidence that the spectral tolerance of this nanostructured anti-reflective coating results from a succession of maxima in the spectrum of different propagative orders, the lowest orders being preponderant in the red part of the spectrum, the highest orders in the blue part.

To conclude, we report the design, fabrication and optical characterization of high performant anti-reflective coatings made of thin nanostructured silicon films. The nanostructuring is made of square silicon pillars 140 nm in width and a square lattice of pitch 240 nm. This design does not require additional thin film coatings. The wide spectral tolerance over the visible spectrum is associated with a very weak dependence on light polarization and to a quasi-omnidirectional light absorption by the structured silicon wafer.

## Methods

### Numerical calculations

The numerical modelling of the structure is performed with a numerical home-made code based on the Fourier Modal Method^39^. This numerical code includes the S-Matrix algorithm to avoid numerical contaminations during the integration process^40^ and the correct rules of factorization of the Fourier series^41^. A detailed description of this method can be found in ref. 42. The optimization of the parameters (period *p*, bumps height *h* and width *w*) is performed through a parametric study under normal incidence (*θ* = 0°). The refractive index of silicon is taken from^43^. The convergence of the numerical method with respect to the number of Fourier coefficients was checked and shows that enough numerical precision is reached for 25 Fourier orders (from −12 to 12) in each direction. When dealing with lossy semi-infinite media, as the substrate made of Si, the transmission efficiency must be calculated as the ratio of the Poynting vector flow in a surface above and under the nanostrutured layer. This can be done by calculating the Fourier components of the field above and under the pillars, *i.e.* at *z* = 0^−^, *z* = *h* + 42.

### Fabrication

**Figure 6:**
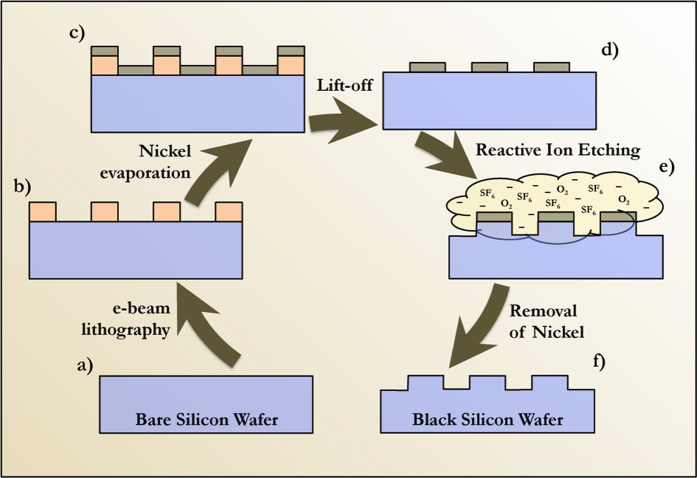
Schema of the fabrication process followed for the etching of thin silicon pillars on the surface of a silicon wafer.

1 cm × 1 cm square samples are cleaved from a 2″ single side polished single crystal silicon wafer (from SPS, Belgium) (Fig. 6a). The samples are orientated in the (100) direction, 250–300 *μ*m thick p-type (boron), and with a resistivity of 1–20 ohm.cm. The samples are cleaned in an ultrasonic bath, first 5 min in acetone and then 5 min in propan-2-ol (IPA). The samples are then rinsed with deionized water and dried under pure nitrogen flow. The cleaned samples are let 10 min at 150 °C in an Oxygen/Argon plasma (Nanoplas, France) in order to increase the wettability and the adhesion of the sensitive resist (Poly(methyl methacrylate), PMMA). The e-beam resist (PMMA 950 K at 2% in ethyl-lactate, AR-P 679 from Allresist GmbH, Germany) is spin-coated at 4000 rpm for 60 sec. The resist is annealed for 10 min at 170 °C to relax stresses and to evaporate residual solvents. The polymer thickness is controlled at 65 nm by a profilometer (Dektak, Bruker Corporation, Germany). E-beam lithography is performed with a Pioneer system (Raith, Germany) in order to create the mask patterns (Fig. 6b), accordingly to the design optimized by numerical simulations. The targeted shape is a square array with 140 nm for the width and 240 nm for the period (and 100 nm for the gap). The write field used for the lithography is a square with 500 *μ*m for the width. A large write field allows for the patterning of large areas with low stitching aberrations. The electron microscope working distance used is 5.8 mm. An acceleration voltage of 20 kV with an aperture of 7.5 *μ*m gives a current between 15 and 22 pA. The dose is chosen to be around 150 *μ*C.cm^−2^ for this configuration. The development of the resist is done by a solution of methyl-isobuthyl-ketone (MIBK) in IPA during 60 sec. The resist is then rinsed in IPA for the same time and finally dried under nitrogen flow. The PMMA resist is positive and the exposed areas are thereby removed. The metallic mask is evaporated under vacuum by thermal effects of a solid nickel source (Auto 306, Edwards vacuum, England) (Fig. 6c). The thickness of the nickel layer is 20 nm. The thickness is monitored during the evaporation by a Quartz microbalance and controlled after the evaporation by a mechanical profilometer. Nickel is chosen for its high resistance to the fluorinated dry etching. The remaining resist with the thin layer of nickel on the top, is removed during the lift-off process (Fig. 6d). The samples are immerged in acetone for a few hours, and then rinsed in ethyl-lactate to remove the last residues of PMMA. The samples are then dried under nitrogen flow.

Silicon dry etching through the nickel mask is performed in a Reactive Ion Etching (RIE) chamber (Plassys, France) (Fig. 6e). We used multistep etching in a gas mixture (SF_6_O_2_) to etch silicon. The RIE set-up uses a capacitive coupled plasma allowing both physical and chemical etching. This combination allows to control anisotropy and fine tuning of the vertical etch depth. Each etching cycle is composed by a first step in SF_6_ – O_2_ gas mixture followed by a step of a pure O_2_ plasma. This oxygen plasma protects the vertical walls in order to increase the anisotropy. From 2 to 4 cycles are used to etch the silicon. The fluorinated plasma creates a chemically stable compound (Nickel(II) fluoride) in the contact of the nickel mask^44^. This mask is not etched by the RIE process. On the other hand, the reaction between the fluorinated plasma radicals and the silicon creates volatile compounds; the silicon is so etched at the rate of 30–50 nm/cycle. The RF power (at 13.56 MHz) is 120 W for the SF_6_ – O_2_ steps (autopolaris voltage: ≈550 V), and 15 W for the pure O_2_ steps. The duration of one cycle is typically 6–9 s. The nickel mask is removed by a home-made acid aqueous solution of FeCl_3_ (30% FeCl_3_ +3% HCl) (Fig. 6e). At room temperature, the etching nickel rate is 10 nm/min. The sample is then rinsed by deionized water and dried under nitrogen flow.

### Optical characterization

The anti-reflective coatings are characterized in a home-made confocal microscope. Light is emitted by a Quartz - Tungsten - Halogen white lamp injected in an optical fiber of diameter 105 *μ*m and collected by a parabolic mirror in order to homogenize the light spot. The final diameter of the beam is 5 mm and it illuminates the sample with a tunable angle *θ*. Finally a Glan-Thompson Polarizer is placed between the parabolic mirror and the sample to tune the polarization of the incident beam (polarization *s* and *p*). The samples are placed at the center of a goniometer. Besides the rotation angle of the goniometer denoted *θ*, the sample can also be independently rotated to collect the reflected light by the microscope objective with an angle 2*θ* between the incident and reflected light. The reflected light is collected by a ×20 microscope objective with a long working distance (20 mm) (Mitutoyo M Plan NIR, Japan). The image is shaped by a lens tube and separated by a beam splitter. 30% of light energy illuminates a CCD camera, the remaining part is coupled with an optical fiber located in the confocal image plane to the sample. The optical fiber is plugged to a spectrometer (Isoplane 160, Princeton Instruments) to analyze the reflected light. The collected area is a circle of 5 *μ*m diameter normal to the sample.

Three spectra are measured for each angle: one background (Bcgd) with a screen in a front of the optical fiber coupled with the spectrometer, one spectrum (I) for bare or patterned silicon, and one spectrum (I_0_) with a silver mirror of reflectivity *R*_*mirror*_(*θ*, *λ*). The reflective spectrum is thereby defined as: 
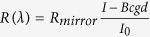
.

## Additional Information

**How to cite this article**: Proust, J. *et al.* Optimized 2D array of thin silicon pillars for efficient antireflective coatings in the visible spectrum. *Sci. Rep.* **6**, 24947; doi: 10.1038/srep24947 (2016).
